# Pathologic Inflammation in Malnutrition Is Driven by Proinflammatory Intestinal Microbiota, Large Intestine Barrier Dysfunction, and Translocation of Bacterial Lipopolysaccharide

**DOI:** 10.3389/fimmu.2022.846155

**Published:** 2022-05-26

**Authors:** Grace T. Patterson, Elvia Y. Osorio, Alex Peniche, Sara M. Dann, Erika Cordova, Geoffrey A. Preidis, Ji Ho Suh, Ichiaki Ito, Omar A. Saldarriaga, Michael Loeffelholz, Nadim J. Ajami, Bruno L. Travi, Peter C. Melby

**Affiliations:** ^1^Division of Infectious Diseases, Department of Internal Medicine, University of Texas Medical Branch, Galveston, TX, United States; ^2^Department of Microbiology and Immunology, University of Texas Medical Branch, Galveston, TX, United States; ^3^Center for Tropical Diseases, University of Texas Medical Branch, Galveston, TX, United States; ^4^Division of Gastroenterology, Hepatology, & Nutrition, Department of Pediatrics, Baylor College of Medicine and Texas Children’s Hospital, Houston, TX, United States; ^5^Department of Pathology, University of Texas Medical Branch, Galveston, TX, United States; ^6^The Alkek Center for Metagenomics and Microbiome Research, Department of Molecular Virology and Microbiology, Baylor College of Medicine, Houston, TX, United States

**Keywords:** malnutrition, inflammation, microbiota, lipopolysaccharide, intestinal barrier

## Abstract

Acute malnutrition, or wasting, is implicated in over half of all deaths in children under five and increases risk of infectious disease. Studies in humans and preclinical models have demonstrated that malnutrition is linked to an immature intestinal microbiota characterized by increased prevalence of Enterobacteriaceae. Observational studies in children with moderate acute malnutrition (MAM) have also observed heightened systemic inflammation and increased circulating bacterial lipopolysaccharides (LPS; endotoxin). However, the mechanisms that underpin the systemic inflammatory state and endotoxemia, and their pathophysiological consequences, remain uncertain. Understanding these pathophysiological mechanisms is necessary to design targeted treatments that will improve the unacceptable rate of failure or relapse that plague current approaches. Here we use a mouse model of MAM to investigate the mechanisms that promote inflammation in the malnourished host. We found that mice with MAM exhibited increased systemic inflammation at baseline, increased translocation of bacteria and bacterial LPS, and an exaggerated response to inflammatory stimuli. An exaggerated response to bacterial LPS was associated with increased acute weight loss. Remarkably, intestinal inflammation and barrier dysfunction was found in the cecum and colon. The cecum showed a dysbiotic microbiota with expansion of Gammaproteobacteria and some Firmicutes, and contraction of Bacteroidetes. These changes were paralleled by an increase in fecal LPS bioactivity. The inflammatory phenotype and weight loss was modulated by oral administration of non-absorbable antibiotics that altered the proportion of cecal Gammaproteobacteria. We propose that the heightened inflammation of acute malnutrition is the result of changes in the intestinal microbiota, intestinal barrier dysfunction in the cecum and colon, and increased systemic exposure to LPS.

## Introduction

Acute malnutrition affects 50.5 million children under the age of five worldwide (1). Children with severe acute malnutrition (SAM) are nine times more likely than healthy children to die, and those with moderate acute malnutrition (MAM) are three times more likely (2). Much of this increased risk of death is due to increased risk and severity of infectious disease, which also contributes to the development and perpetuation of malnutrition (3).

Despite the global importance of childhood malnutrition, little is know about the pathophysiological mechanisms beyond dietary protein and micronutrient deficiencies that underpin its development. Inadequate water, sanitation and hygiene (WASH) is commonplace in regions of the world where childhood malnutrition is prevalent. However, interventions to improve WASH have had little impact on malnutrition rates at the current level of implementation (4–6). A number of studies describe immunological deficits in children with malnutrition (reviewed in (3, 7)). However, little is understood about the mechanistic links between immune deficits and malnutrition, and whether nutritional interventions can effect recovery of immune function (3). Collectively, these observations suggest that contributors to growth faltering and recovery are not well understood and remain untargeted (4–6). Conventional treatments for MAM and SAM, which are largely focused on growth metrics, have high failure and relapse rates (8), probably because they fail to address the pathophysiological underpinnings of malnutrition (4, 8).

Intestinal dysbiosis is both a contributor to, and consequence of, malnutrition. Studies of children with SAM and MAM show that malnourished children have “immature” intestinal microbiota compositions that do not develop along normal timelines (9–11). The immature microbiota is characterized by low diversity, increased prevalence of aerotolerant species from Enterobacteriaceae (9, 12, 13), and decreased prevalence of beneficial commensal species (14). This stunted microbiota hampers energy harvest and allows enteropathogens to flourish, driving impaired barrier function and intestinal inflammation (10, 12). Transplantation of fecal microbiotas from children with SAM into germ-free mice demonstrated that the combination of a nutrient deficient diet and malnourished microbiota can cause weight loss, increased intestinal permeability, and intestinal damage (11). A study of bacterial species targeted by IgA in children with SAM and enteropathy suggested that members of Enterobacteriaceae were drivers of weight loss and disrupted intestinal barrier function (15). Preclinical studies have shown that the contribution of a nutrient-deficient diet and altered intestinal microbiota to growth faltering is amplified when the host is repeatedly exposed to a collection of non-pathogen commensal bacteria (14, 16). There are parallels between malnutrition-driven dysbiosis and the dysbiosis observed in aging and disorders such as colitis, obesity, metabolic syndrome, and Environmental Enteric Dysfunction (EED) (17). The latter is a disorder of small intestine damage characterized by villus blunting or atrophy, inflammatory cell infiltration, and hyperplasia of small intestine crypts that promote increased permeability and malabsorption. EED occurs in children from low-resource settings with poor sanitation and microbial contamination of food and water. EED is causally related to components of the duodenal microbiota (18) and leads to growth faltering and stunting (chronic malnutrition), but it is distinct from acute malnutrition. The link between dysbiosis and systemic inflammation has been better characterized in these disorders and may suggest potential mechanisms for the increased systemic inflammation observed in MAM. In each of these disorders there is an increased proportion of intestinal gram-negative bacteria, leading to leakage of LPS and endotoxemia (19–22). Thus, we aim to investigate pathways from these related disease profiles in our effort to identify MAM-related systemic inflammation and how it relates to translocation, intestinal immunity, and microbiota.

The inflammatory response is energetically costly for malnourished children and diverts already scarce nutrients to acute phase and immune protein production (23). Observational studies in children with SAM (24) and MAM (25) have linked malnutrition, intestinal damage, and elevated circulating LPS. However, the mechanisms that underpin these findings are obscure.

In this study, we used a mouse model of childhood MAM that is initiated by weaning mice to a diet deficient in protein, energy, and selected micronutrients (iron and zinc) to investigate the links between intestinal microbiota, intestinal barrier function, and systemic inflammation. We found that mice with MAM exhibited increased systemic inflammation at baseline, increased translocation of bacteria and bacterial LPS, and an exaggerated response to inflammatory stimuli. This exaggerated response had the pathophysiologic consequence of increased weight loss. Strikingly, intestinal inflammation and barrier dysfunction was primarily localized to the cecum and colon rather than the ileum. The cecum showed a dysbiotic microbiota with expansion of Gammaproteobacteria and some Firmicutes, and contraction of Bacteroidetes. These changes were paralleled by an increase in fecal LPS bioactivity. The inflammatory phenotype and weight loss was modulated by oral administration of non-absorbable antibiotics that influenced the proportion of these phyla. We propose that the heightened inflammation of acute malnutrition is the result of changes in the intestinal microbiota, intestinal barrier dysfunction in the cecum and colon, and increased systemic exposure to LPS.

## Materials and Methods

### Mice and Diets

Weanling female BALB/C mice (3-4 weeks of age) were obtained from Envigo. Mice were grouped in cages of 5 and had free access to water. Mice were weaned to either a standard mouse chow (Teklad diet number 99103) or an isocaloric but protein, iron, and zinc deficient malnourished diet (Teklad diet number 99075) for 28 days. Mice were pair-fed every 48-72 hrs and weighed every 7 days. Malnourished (MN) mice received 90% volume by weight of food that control (normal diet) mice did. This was calculated by determining g/mouse/day for the previous feeding period for well-nourished mice and then determining the necessary amount per cage for MN mice.

### Intradermal Challenge With Inflammatory Stimuli

Well-nourished and malnourished mice (5 mice per group) were injected intradermally in the skin of the dorsal foot with 20 µL of either LPS (10 µg) (*Escherichia coli*, L4391-1MG; Sigma-Aldrich, St. Louis, MO), lipotechoic acid (LTA; 50 µg) (*Staphylococcus aureus*, Sigma-Aldrich, L2515-5MG), or PBS (control). Mice were euthanized 24 hours post-injection *via* CO_2_ inhalation and cervical dislocation.

### Systemic LPS Challenge

Mice were weaned to either a well-nourished or malnourished diet as previously described. After 28 days on the diet, mice were injected intraperitoneally with 4 mg/kg of LPS (*Escherichia coli* O55:B5, Sigma-Aldrich, L2880) in PBS. Mice were given food and water ad libitum and monitored hourly. At six hours post injection, mice were weighed. Mice were weighed again at 24 hours post injection and blood was collected *via* cardiac puncture under isofluorane anesthesia and mice euthanized.

### Tissue Expression of Cytokines

Tissue from skin (site of injection), ileum, cecum and colon was removed and stored in RNAlater Stabilization Solution (Thermofisher) at -80°C until RNA extraction and quantification by qPCR. Samples were thawed on ice and 30 mg or less of each sample (skin, ileum, and/or liver) was removed from RNAlater, homogenized in lysis buffer (Qiagen) + B-mercaptoethanol (Sigma) *via* either tissue homogenizer (IKA Utra-Turrax T18 Homogenizer) or bead mill (VWR 4-Place Mini Bead Mill Homogenizer), and spun down to pellet remaining tissue. RNA was extracted from the supernatant (RNeasy, Qiagen) and treated with DNAse (Turbo DNAse, Ambion). All RNA samples were quantified using a Thermo Scientific NanoDrop™ Spectrophotometer and stored at -80°C. cDNA was generated from each sample using the High Capacity cDNA Reverse Transcription kit (Applied Biosystems) (Veriti 96 Well Thermal Cycler, Applied Biosystems). Cytokine mRNA was quantified using 20-40 ng cDNA, SYBR Green Master Mix (ThermoFisher), and primers (see **Supplemental Table 1**) on a Viia 7 Real-Time PCR System (Applied Biosystems). CT values were used to calculate fold change in comparison to the mean of naïve samples from control mice.

### TNF Production by Peritoneal Macrophages

Resident peritoneal cells were recovered from control and MN mice in by peritoneal lavage with 5 mL chilled RPMI with 10% HIFBS, 50 µM B-mercaptoethanol (Sigma), washed, and viable cells were counted adjusted to 0.5 × 10^6^/mL. The cell suspensions were plated in 1 ml aliquots in a 24 well plate and allowed to adhere for 2 hours at 37°C and 5% CO_2_. Cells were washed twice with 2% RPMI to remove non-adherent cells. Cells (4 replicates per stimulus) were then stimulated with either 20 µg/mL LTA, 10 ng/mL LPS, or 2% RPMI as control. After 4 hours incubation at 37°C and 5% CO_2_, 2 mL of supernatants were collected and stored at -80°C until analyzed by ELISA. Supernatants from macrophage stimulation were thawed on ice and centrifuged at full speed for 10 minutes to pellet any remaining cells. Samples were diluted 1:1 in Standard Diluent Buffer (Invitrogen) prior to cytokine level measurement *via* ELISA (Invitrogen). Plate was read on a FLUOstar Omega plate reader (BMG Laboratories) and OMEGA software (BMG Laboratories) was used to generate a four-parameter logistic curve to calculate protein concentrations.

### Bacterial Translocation

Spleen, liver, and mesenteric lymph nodes (MLN) were used to determine bacterial burden. The liver was cut into thirds and the anterior portion of the middle section was collected. The spleen was cut in half and the anterior half was collected. Spleen (10-35 mg), liver (40-90 mg), and MLN (3-12 mg) samples were stored in a 10 volumes of PBS (e.g. 50 mg tissue, 500 µl PBS) in a 2 ml Eppendorf tube. Samples were homogenized in the collection tube with a rubber pestle. Each sample was serially diluted out to 10^-6^ in PBS, generating a range of tissue concentrations from 10 mg/µl to 10 ng/µl. 50 µl aliquots of each dilution were plated on Brain Heart Infusion (BHI) Agar (Sigma-Aldrich) plates and incubated aerobically and anaerobically at 37°C. Plates were incubated 3 days and colonies counted on both days 2 and 3. Colony counts and dilution values were used to calculate CFU/g for each sample.

### Identification of Bacteria by MALDI-TOF Mass Spectrometry

Random colonies of various morphologies were collected from aerobic and anaerobic cultures of MLN, liver and spleen grown on BHI agar. MALDI-TOF MS (bioMérieux, Inc., Durham, NC) was performed according to the manufacturer’s protocol. Briefly, an isolated colony was directly spotted on a disposable slide using a plastic loop and overlaid with α-cyano-4-hydroxycinnamic acid matrix solution. Captured spectra were analyzed using the bioMérieux Vitek MS IVD database version 2.0. Vitek MS analysis produces a confidence value, reported as a percentage up to 99.9%. The identification criteria used were those recommended by the manufacturer. Confidence values of ≥60% indicated species level identification. Obtaining a single genus, regardless of the confidence value, was considered an acceptable genus level of identification.

### Histology

Small intestine (ileum) and colon were removed, opened longitudinally, processed as Swiss rolls, and fixed for 48 hours in 10% buffered formalin. Fixed tissue was paraffin embedded and sectioned (5 μm) for hematoxylin and eosin (H&E) staining. Blinded histopathological examination of stained sections was performed by two independent observers. For ileal sections, villus length was measured from the base to the tip of the villus. For colon sections, crypt length was measured from the base of the crypt to the mucosal surface. Four villi and four crypt lengths were measured for each tissue section and the scores averaged. The number of inflammatory cells in the lamina propria of the ileum and colon were obtained using a 40X objective. The number of cells per 15 crypts were counted and averaged for 4 non-contiguous areas (26). Images were obtained at 40X using a brightfield microscope (Nikon Eclipse 80i) and analyzed using NIS-Elements Basic Research (Nikon V 3.2).

### *In Vivo* and *Ex Vivo* Measurement of Paracellular Intestinal Permeability

*In vivo:* Control and MN mice were fasted overnight and underwent oral gavage with a mixed suspension of 100 mg/mL of 4 kDA FITC-dextran (46944, Sigma) and 100 mg/mL of 70 kDA rhodamine B isothiocyanate-dextran (R9379, Sigma) in PBS corresponding to 600 mg/kg body weight of each dextran. After one hour of transit time to allow the suspension to reach the ileum (27), blood was collected *via* cardiac puncture under isofluorane anesthesia. Blood was centrifuged at 2000g for 10 minutes at 4°C and serum collected. Centrifugation was repeated once and serum was diluted 1:1 in PBS. Diluted serum and mixed dextran standards were plated on a 96 well plate in duplicate and read on a FLUOstar Omega plate reader (BMG Laboratories). An excitation of 485 nm and emission of 520 nm was used to measure FITC-Dextran concentration and an excitation of 544 nm and emission of 590 nm was used to measure rhodamine B isothiocyanate-dextran concentration. OMEGA software (BMG Laboratories) was used to generate a linear regression fit standard curve. For intracolonic instillation, 600 mg/kg 4 kDa FITC-dextran (Sigma) was delivered with a 2.5 cm flexible plastic tube with silicone tip in a volume of 100 uL PBS. One hour post-instillation blood was obtained by retro-orbital bleeding using heparinized capillary tubes or the animals were euthanized and liver tissue harvested. Fluorescent dextran was quantified in plasma or liver tissue by fluorometry. *Ex vivo:* Intestinal permeability was quantified in a P2300 Ussing chamber system (Physiologic Instruments, San Diego, CA) as described previously (28). Briefly, full-thickness sections of cecum and colon were harvested and flushed with KBR buffer (in mM: 115 NaCl, 1.2 CaCl_2_, 25 NaHCO_3_, 2.4 K_2_HPO_4_, 0.4 KH_2_PO_4_, 1.2 MgCl_2_, 10 glucose). Each segment was opened along its mesenteric border, rinsed, and mounted on a slide with an exposure area of 0.10 cm^2^. The mucosal and serosal chambers each were filled with 5 mL KBR buffer maintained at 37°C and infused with 95% O_2_/5% CO_2_ gas. Permeability was assessed by quantifying flux of 4 kDa FITC-dextran (Sigma). After an equilibration period of 10 min, FITC-dextran was added to the mucosal side of the chamber to a final concentration of 2.2 mg/mL and sampled from the serosal side after 0, 30, 60, 90, and 120 min. The fluorescent intensity of each sample was measured by a Cytation5 Imaging Reader (BioTek Instruments, Winooski, VT).

### Endotoxin Quantification

Serum was isolated from control and MN mice as described previously. Endotoxin was quantified using the HEK-Blue LPS Detection Kit 2 (InvivoGen, San Diego, California, USA) as per manufacturer’s protocol. Samples were diluted 1:3 in assay diluent prior to quantification.

### Isolation of Total Fecal LPS

Total LPS was isolated from fecal samples according to the method of d’Hennezel, et al, with slight modifications (29). Approximately 500 mg of fecal material was homogenized into 1 ml of HyPure cell culture grade endotoxin-free water (HyClone). Material was processed using a bead beating Mini Bead Mill (VWR) instrument with XXTuff reinforced microvials and 3.2mm Stainless Steel Beads (Biospec Products, Bartlesville, OK, USA). The resulting fecal slurry was allowed to settle for 5 min, allowing large particles to settle, the supernatant (~1 mL) was collected in a new vial and centrifuged for 3 min at 14,000 rpm RT. The pellet was processed according to instructions provided in the LPS Extraction Kit, a Phenol-based Lipopolysaccharide Purification supplied by Bulldog-Bio Inc (Portsmouth, NH). Briefly, pellets were resuspended and vortexed in 1 ml of lysis buffer, and then mixed with chloroform. Following incubation at RT for 5 min, suspensions were centrifuged 10 min. The supernatant was transferred to a new tube and two volumes of purification buffer was added and incubated for 10 min. The resulting pellet was washed with 70% ethanol and resuspended in 10 mM Tris-HCl buffer (pH 8.0). Bioactive fecal LPS was quantified as described above.

### Microbiota Analysis

Cecal contents and mesenteric lymph nodes (MLN) were collected from control and MN mice and transported on icepacks to the Alkek Center for Metagenomics and Microbiome Research at Baylor College of Medicine for 16S rRNA sequencing and analysis. DNA was extracted from cecal contents with the PowerSoil DNA Isolation kit (MoBio) and from MLN with the UltraClean Tissue and Cells DNA Isolation kit (MoBio). DNA underwent 16Sv4 PCR (30) followed by MiSeq 2x250 bp sequencing and the CMMR-generated 16S analysis pipeline.

### Antibiotic Treatment

Mice were placed on either a control or nutrient-deficient diet as previously described. After 14 days on the diet antibiotics were administered in the drinking water for the final 14 days of the diet. Mice were given either 0.7 mg/mL of vancomycin hydrochloride (*Streptomyces orientalis*, Sigma Aldrich, V8138), 7,500 U/mL colistin sulfate salt (Sigma Aldrich, C4461), or normal water as a control. Water bottles were replaced every 3-4 days. Actual dosages calculated from average water consumption were approximately 5 mg/day of vancomycin and 33,750 U/day colistin. After 14 days on antibiotics, mice were euthanized and skin, blood, and cecal matter were collected and stored at -80°C. Liver, spleen, and MLN were also collected and used to quantify bacterial burden as described above. mRNA was isolated from skin samples and quantified as listed previously. To determine the microbiota composition in the antibiotic-treated mice, DNA was isolated from cecal matter using the QIAamp DNA Stool Mini Kit (QIAGEN) according to manufacturer’s protocol and shipped on dry ice to LCSciences (Houston, Texas, USA) for 16S rDNA V3+V4 sequencing and analysis. Bacterial DNA was amplified with primers targeted to the V3 and V4 regions of 16S rDNA. Sequencing adaptors and barcodes were added in further amplification steps and the prepared library was sequenced on the MiSeq platform. Resultant paired-end reads were merged into tags and then grouped into clusters that represent a single operational taxonomic unit (OTU) (minimum 97% sequence similarity). Analysis of OTUs were conducted including measures of alpha diversity, beta diversity, and taxonomy annotation. Databases for taxonomy analysis included RDP (version date 2016.9.30) and NT-16S (version date 2016.10.29). Z scores for the proportions of genera were calculated using a Wilcoxon rank sum test.

### Statistics

Comparisons between 2 groups were evaluated with two tail Mann-Whitney U test for non-parametric data or two tail unpaired t test for normally distributed data. Comparisons between more than 2 groups were evaluated with Kruskal-Wallis for non-parametric data or ANOVA for normally distributed dated with *post hoc* correction for multiple comparisons (Bonferroni or Tukey). Outliers were identified *via* the ROUT method with coefficient Q 1% (31) and removed from analysis where appropriate. All analyses were conducted using GraphPad Prism 8 for Mac OS X (Graphpad Software, San Diego California USA). In general, the data are presented in graphs as the mean and standard deviation of the group. When the data are skewed they are presented as the median with interquartile range, which is noted in the figure legends.

## Results

### Malnourished Mice Exhibit Increased Baseline Inflammation and an Exaggerated Response to Inflammatory Stimuli

To explore the effects of MAM on inflammation, we used a mouse model of combined macro-nutrient (protein) and micro-nutrient (iron and zinc) deficiency that mimics the diet that often leads to childhood MAM (32, 33). Experimental diets were identical except that they were made to be isocaloric by increasing the carbohydrates in the malnourished diet to account for the reduced protein content. The mice were pair-fed and the MN mice received 10% less chow than control mice, generating a modest energy deficit. At time of randomized weaning to the experimental diets, mice in the control and MN groups were indistinguishable by weight (mean 14.0g and 13.5g, respectively) (**Figure S1**). Mice on the control diet reached their adult weight (mean=19.35 g) by day 28 of the diet and gained 38% of their starting bodyweight. MN mice at day 28 averaged 12.5 g and lost 8% of their starting bodyweight.

**Figure 1:**
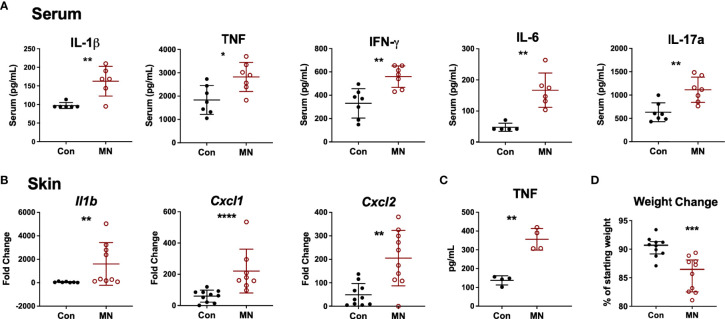
Malnourished mice exhibit heightened baseline inflammation and an exaggerated inflammatory response to bacterial ligands. **(A)** Inflammatory cytokines in serum of control (Con) or malnourished (MN) mice determined by Luminex assay (n=8-9 mice per group). **(B)** Cytokine mRNA expression determined by qRT-PCR in skin from the dorsal foot of control and MN mice (n=4-5 mice per group) 24 hours post-injection with 10 µg LPS in 20 µL of PBS or an equal volume of PBS alone. Fold-change determined by ratio of expression of target mRNA in LPS-administered vs. PBS. Data shown are pooled from 2 independent experiments. **(C)** TNF concentration determined by ELISA in supernatants of peritoneal macrophages isolated from control and MN mice cultured for 4 hours with or without 10 ng/mL LPS or 20 µg/mL lipotechoic acid (LTA). TNF-α levels were below the limit of detection in the supernatant of untreated cells. **(D)** Weight change in control and MN mice that were challenged intraperitoneally with 4 mg/kg LPS. Shown is the percentage of starting weight at 24 hours post-challenge. Data presented is pooled from three independent experiments. (*p < 0.05; **p < 0.01; ***p < 0.001 ****p < 0.0001).

At baseline, MN mice were found to have increased serum proinflammatory cytokine levels (IL-1β, TNF, IFNγ, IL-6 and IL-17A) compared to control mice (**Figure 1A**). To investigate the response to an inflammatory stimulus, we injected bacterial lipopolysaccharide (LPS; from gram-negative bacteria) or lipotechoic acid (LTA; from gram-positive bacteria) intradermally in the dorsal skin of the foot of MN and control mice. Injection of sterile PBS was used as control. Twenty-four hours post LPS injection, MN mice had increased dermal expression of the pro-inflammatory cytokine *Il1b*, the neutrophil chemoattractants *Cxcl1* and *Cxcl2*, but not the monocyte chemoattractant *Ccl2* compared to controls. (**Figure 1B**). Interestingly, MN mice also showed a small but significantly increased cytokine response to sterile tissue injury (intradermal PBS) which served as the control for the LPS and LTA injected mice (**Figure S2A**). The dermal response to LPS (**Figure 1B**) was considerably greater than the response to LTA (**Figure S2B**), which showed little difference between control and MN mice. Peritoneal macrophages isolated from MN mice and stimulated with LPS or LTA produced greater quantities of TNF than peritoneal macrophages isolated from control mice (**Figure 1C**). The exaggerated response to an inflammatory stimulus in the MN mice had pathophysiological consequences. MN mice injected intraperitoneally with a sub-lethal dose of LPS lost 13.5% of their starting body weight, while control mice lost 9.3% of their starting weight (*p* = 0.0007) (**Figure 1D**). Collectively, these data indicate that malnutrition results in increased basal inflammation, an exaggerated inflammatory response to sterile tissue injury or bacterial products, and greater pathophysiological impact (weight loss) following exposure to bacterial LPS.

### Malnourished Mice Show Increased Translocation of Aerobic Bacteria and Bacterial LPS

**Figure 2:**
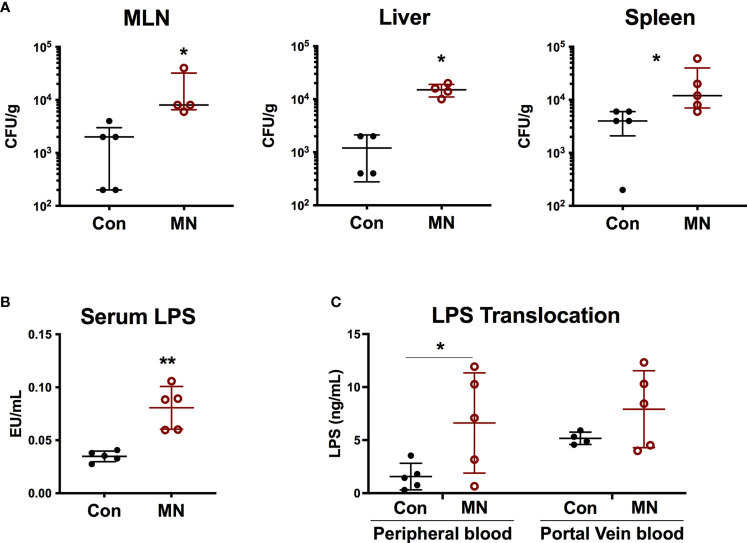
Malnutrition promotes translocation of bacteria and bacterial LPS. **(A)** Quantification of culturable aerobic bacteria in the mesenteric lymph nodes (MLN), liver, and spleen of control (Con) and malnourished (MN) mice. Live bacteria were quantified by plating tissue homogenates on BHI agar (n=4-5 mice per group). Data are presented as the median with interquartile range. **(B)** Measurement of bioactive LPS in serum from peripheral blood collected from control and MN mice (n=5 mice per group). Determined by HEK-blue TLR4 bioassay. **(C)** Control and MN mice were administered 0.1 mg/kg body weight of Alexa Fluor488-labeled LPS by oral gavage. Two hours after administration, blood was taken from heart and portal vein. Fluorescence (Ex: 485 nm/Em: 520 nm) in sera was measured by fluorometer to quantify LPS concentration (ng/mL AF488-LPS). (*p < 0.05; **p < 0.01).

We hypothesized that the heightened baseline inflammation and response to bacterial stimuli observed in MN mice may be a result of exposure to bacteria or bacterial products translocating from the gut. Indeed, we observed increased numbers of culturable aerobic bacteria in the MLN, spleens, and livers of MN mice compared to control mice (**Figure 2A**). There was no increase in numbers of culturable anaerobic bacteria in the mesenteric lymph node (MLN), spleens, and livers of MN mice (**Figure S3A**). A random sample of aerobic and anaerobic bacterial colonies from the spleens and livers of both control and MN mice were identified *via* mass spectrometry. Ten of 67 colonies identified were gram-negative, arising from three different species (eight *Escherichia coli*, one *Enterobacter amnigenus*, and one *Prevotella buccalis*). The remaining 57 colonies belonged to gram-positive bacteria of phyla Firmicutes and included isolates from genera *Enterococcus* (25)*, Staphylococcus* (25), *Aerococcus* (3), *Streptococcus* (2), *Clostridium* (1)*, and Leuconostoc* (1) (**Figure S3B**). MN mice also had a significantly higher level of serum LPS compared to control mice (**Figure 2B**). The route of translocation of free LPS from the gut to peripheral and portal blood was altered in MN mice. Two hours after oral gavage of mice with fluorescent-labeled LPS, a time in which the bolus would likely have transited into the large intestine, the control mice showed translocation of fluorescent-labeled LPS primarily into the portal vein, however, MN mice showed significantly greater translocation of LPS into the peripheral circulation (**Figure 2C**). The ratio of LPS concentration in peripheral to portal blood was 0.25 in control mice was but 0.87 in MN mice. These data demonstrate that MN mice experience greater systemic exposure to bacteria and bacterial products.

### Malnourished Mice Have Evidence of Impaired Intestinal (Colonic) Barrier Function and Altered Intestinal Inflammation

**Figure 3:**
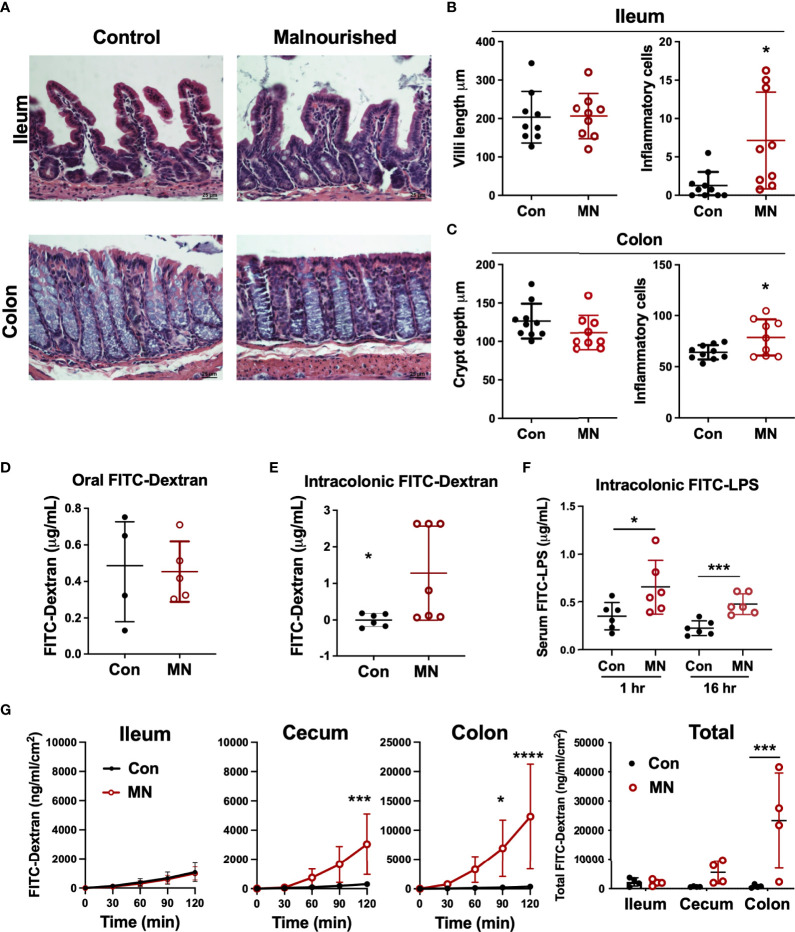
Malnutrition promotes increased inflammation and permeability in the cecum and colon but spares the ileum. **(A)** Histopathology of ileum and colon in malnourished and well-nourished control mice. **(B)** Villus height and number of inflammatory cells in ileum of control (Con) and malnourished (MN) mice. **(C)** Crypt length and number of inflammatory cells in colon of control and MN mice. **(D)** Concentration of 4 kDa FITC-Dextran in serum of control and MN mice 1 hour after oral gavage. **(E)** Concentration of 4 kDa FITC-Dextran in serum of control and MN mice 1 hour after intracolonic delivery. **(F)** Concentration of FITC-LPS in serum of control and MN mice 1 hour and 16 hrs after intracolonic delivery. **(G)** *Ex vivo* paracellular permeability of intestinal epithelium from ileum, cecum, and colon (n=4 samples of each tissue) in control and MN mice over 2 hours diffusion in an Ussing diffusion chamber. Data are expressed as median with interquartile range of concentration over time measured in the basolateral side following delivery of 4 kDa FITC-dextran to the apical side of the intestinal epithelium of the ileum, cecum, and colon, and as composite figure where the data were normalized to the average measurements over all time points. (*p < 0.05; ***p < 0.001; ****p < 0.0001).

**Figure 4:**
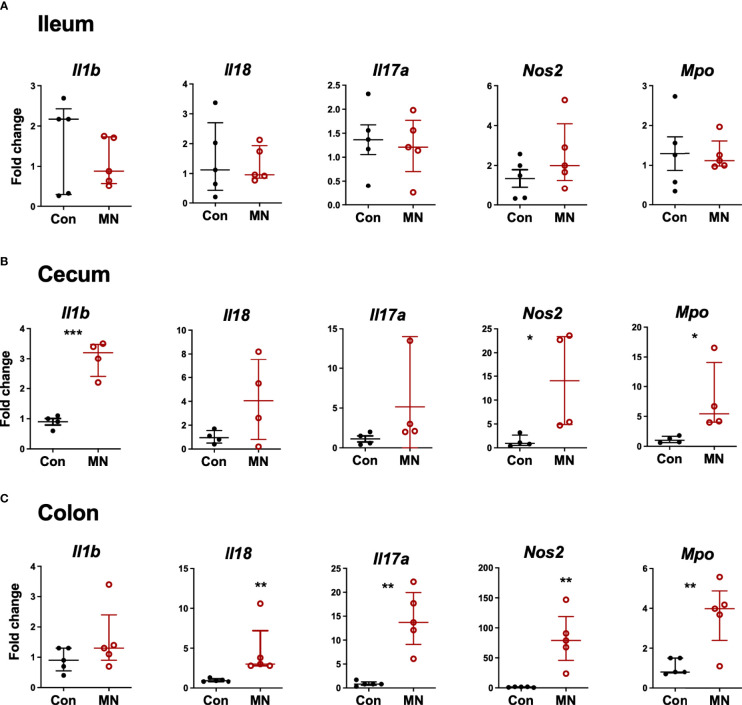
Inflammatory mediator expression in tissue from ileum, cecum and colon. Intestinal tissue from **(A)** ileum, **(B)** cecum, and **(C)** colon was harvested from control and MN mice. mRNA expression of selected inflammatory mediators and markers of inflammation was determined by qRT-PCR. Fold-change of expression in MN mice was determined relative to the mean expression in tissue from control mice. Data are presented as the median with interquartile range. (*p < 0.05; **p < 0.01; ***p < 0.001).

To determine if the increased extra-intestinal bacterial burden and LPS translocation was due to impaired intestinal barrier function, we first examined the histological structure of the ileum, cecum, and colon (**Figure 3A**). We found no significant differences in the villous height in the ileum, but there were increased inflammatory cells in the lamina propria (**Figure 3B**). The colon showed insignificant (p=0.09) reduction in crypt length and increased inflammatory cell infiltration in the lamina propria in MN mice (**Figure 3C**). Oral gavage of low molecular weight (4 kDa) fluorescent dextran showed no difference in serum concentration (paracellular intestinal permeability) at 1 hr post-gavage (**Figure 3D**). In contrast, sampling of serum 1 hr after intracolonic instillation of 4 kDa FITC-dextran revealed increased translocation in MN compared to control mice (**Figure 3E**). Similarly, intracolonic instillation of FITC-LPS led to increased translocation to serum in MN compared to control mice when measured at 1 and 16 hrs after instillation (**Figure 3F**). *Ex vivo* analysis of paracellular permeability of the intestinal epithelium from the ileum, cecum and colon using 4 kD FITC-dextran in an Ussing diffusion chamber revealed no differences in the ileum of MN and control mice, but the cecum and colon of MN mice had significantly increased diffusion of the fluorophore from apical to basolateral side of the epithelium (**Figure 3G**). Collectively these data indicate that impaired barrier function in malnourished mice is localized to the large intestine. Consistent with the evidence that the cecum and colon, but not ileum, have impaired barrier function, we found increased expression of inflammatory cytokines, *Nos2*, and myeloperoxidase (*Mpo*; an indicator of the presence of activated neutrophils) in the cecum and colon of malnourished mice, but not in the ileum (**Figures 4A-C**).

### Cecal Microbiota From MN Mice Is Characterized by Increased Proportions of Firmicutes and Proteobacteria and Decreased Bacteroidetes

**Figure 5:**
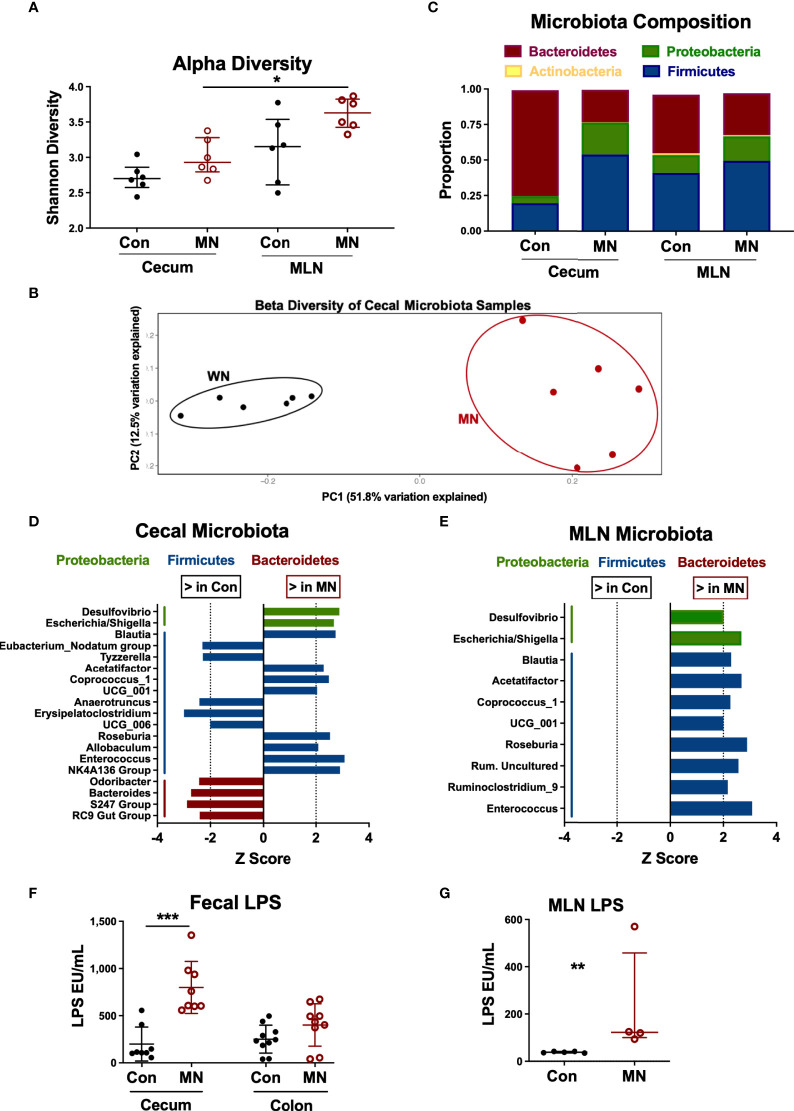
Malnutrition increases proportions of Proteobacteria and Firmicutes and bioactive LPS in cecum and mesenteric lymph node. Microbiota composition determined by bacterial 16S rRNA sequencing of cecal contents and MLN samples from 5 control (Con) and 5 malnourished (MN) mice. **(A)** Alpha diversity for control and MN cecal stool and MLN samples. **(B)** Beta diversity for control and MN cecal content microbiota. **(C)** Average proportions of four most common phyla in cecal stool and MLN samples. **(D, E)** Significant (p < 0.05, Wilcoxon signed-rank test) genus-level differences in microbiota of the cecum **(D)** and MLN **(E)** between control and MN mice. Z score indicates level of variance in proportion between Con and MN mice (i.e. values further from 0 indicate greater difference between groups). **(F)** Total bioactive fecal LPS in contents of cecum and colon of control and MN mice. Determined by HEK-blue TLR4 bioassay. **(G)** Concentration of bioactive LPS in MLN draining the proximal large intestine of control and MN mice. Determined by HEK-blue TLR4 bioassay. Data are presented as the median with interquartile range. (*p < 0.05; **p < 0.01; ***p < 0.001).

The increased bacterial translocation, elevated circulating LPS, and altered intestinal cytokine and chemokine expression observed in MN mice may be due in part to changes in the intestinal microbiota. Therefore, we characterized the bacterial composition of cecal contents and MLN in control and MN mice by 16S sequencing. The microbiota composition of cecal contents from control and MN mice showed no difference in alpha diversity (measure of microbiome diversity within a single sample; **Figure 5A**) but were distinct in beta diversity (measure of microbiome diversity in samples from different environments; **Figure 5B**). In cecal contents there was a decrease in overall proportion of Bacteroidetes (*p* = 0.0065) and an increase in Proteobacteria (*p* = 0.0039) and Firmicutes (*p* = 0.0104) in the MN mice (**Figure 5C**). Differences in the MLN paralleled the cecum, but were not statistically significant. OTUs enriched in the cecal contents of MN mice compared to control mice include two Proteobacteria OTUs (*Escherichia*/*Shigella*, *Desulfovibrio*) and seven Firmicutes OTUs (including *Blautia*, *Roseburia*, *Allobaculum*, and *Enterococcus*) (**Figure 5D**). OTUs reduced in the cecal contents of MN mice included four Bacteroidetes (including *Odoribacter* and *Bacteroides*) and five Firmicutes (**Figure 5D**). All significant OTU-level changes in the MLN of MN mice consisted of an increase compared to control mice; there were no significantly decreased OTUs in the MLN of MN mice (**Figure 5E**). Both Proteobacteria OTUs that were increased in proportion in the MN cecal contents were also increased in the MLN of MN mice. All but two of the Firmicutes OTUs that were increased in the cecal contents of MN mice were also increased in the MLN of MN mice. Thus, control and MN mice have striking differences in the cecal microbiota composition that are replicated in part in the draining MLNs.

While all gram-negative bacteria contain LPS on their surface, there are significant differences in the bioactivity of LPS from different bacteria. LPS from the anaerobic gram-negative members of the order Bacteroidales (e.g. *Bacteroides* spp.) is much less potent than that of *E. coli* and inhibits the TLR-mediated response to *E. coli* LPS (29). Because of the dominance of *Bacteroides* spp. in the intestine under normal homeostatic conditions, the total fecal LPS is immune-inhibitory (29). Thus, we hypothesized that the malnutrition-induced dysbiosis (including increased Proteobacteria and reduced Bacteroidales) would lead to a more bioactive LPS mass in the colon. Indeed, we found that the total fecal LPS from the cecum (*p*=0.001) and colon (not significant; *p*=0.10) from MN mice induced greater TLR4 activation than the total fecal LPS from control mice (**Figure 5F**). Similarly there was greater bioactive LPS in the MLN draining the cecum and upper colon in MN compared to control mice (**Figure 5G**).

### Alteration of the Intestinal Microbiota by Oral Non-absorbable Antibiotics Modulates Inflammation and Weight Loss in Malnourished Mice

**Figure 6:**
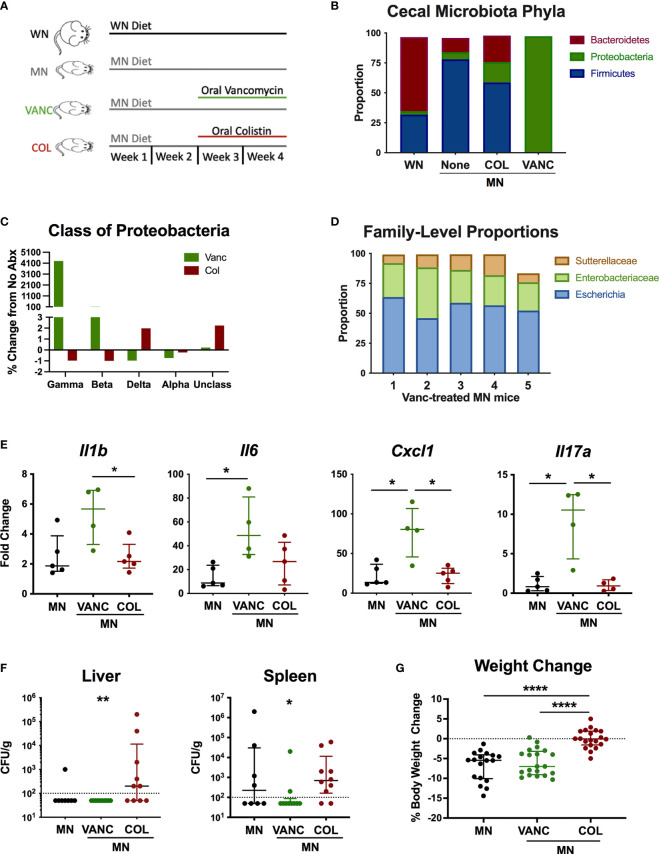
Antibiotic-mediated reshaping of intestinal microbiota modulates bacterial translocation and systemic inflammation. **(A)** Graphic representation of experimental design in which well-nourished (normal diet; WN) and MN mice were given no antibiotic, or MN mice were given non-absorbable vancomycin (VANC) or colistin (COL) in drinking water over last two weeks of the 4-week diet. **(B–D)** Microbiota composition determined by bacterial 16S rDNA sequencing of cecal contents from control (WN) mice, and MN mice treated with no antibiotic (None), oral vancomycin (VANC), or colistin (COL) (n=5 mice per group). **(B)** Data are shown at the Phyla level. **(C)** Data are shown at the Class level as percent change from MN mice that received no antibiotic treatment. **(D)** Microbiota composition for 5 individual vancomycin-treated MN mice shown at the Family level. **(E)** Cytokine mRNA expression determined by qRT-PCR in skin of dorsal foot 24 hrs after intradermal LPS challenge of malnourished mice that had been treated with no antibiotic (MN) or oral vancomycin (VANC) or colistin (COL) (n=5 mice per group). **(F)** Quantification of culturable aerobic bacteria in the liver and spleen of untreated malnourished mice (MN) and malnourished mice treated with oral colistin (COL) or vancomycin (VANC). Live bacteria were quantified by plating tissue homogenates on BHI agar (n=4-5 mice per group). Data are pooled from 2 experiments with 4-5 mice per group and are presented as the median with interquartile range. **(G)** Change in body weight of malnourished mice treated with no antibiotic (MN) or oral vancomycin (VANC) or colistin (COL) in drinking water. Data pooled from 2 experiments with each having 8-10 mice per group. (*p < 0.05; **p < 0.01; ****p < 0.0001).

To determine if antibiotic-mediated change in the intestinal microbiota of MN mice would modulate the systemic inflammation and heightened response to bacterial LPS, we selectively depleted broad bacterial groups by oral administration of non-absorbable antibiotics. For the last 14 days of the 28-day diet, MN mice were given vancomycin (VANC), colistin (COL), or no antibiotic in their drinking water (**Figure 6A**). These antibiotics have negligible systemic absorption following oral administration so would have minimal direct impact on bacteria outside the gastrointestinal tract. VANC is selectively active against aerobic and anaerobic (34) gram-positive bacteria, and COL is selectively active against gram-negative aerobic bacilli (primarily Gammaproteobacteria) (35). To determine the effects of the oral antibiotics, we determined the microbiota composition of cecal contents of well-nourished (WN) and MN mice given no antibiotic, and MN mice given either VANC or COL. Surprisingly, COL-treated MN mice showed an increased proportion of the phylum Proteobacteria compared to non-treated MN mice (**Figure 6B**). However, this increase was due to increases in Deltaproteobacteria and unclassified Proteobacteria (**Figure 6C**) but proportions of Gammaproteobacteria and Betaproteobacteria were reduced from 0.02 and 0.096% respectively in non-treated MN controls to < 0.01% in COL-treated MN mice (**Figure 6C**). COL-treated MN mice also had expansion of Bacteroidetes and reduction of Firmicutes in comparison to MN controls (**Figure 6B**). Conversely, the cecal microbiota of VANC-treated mice consisted of >95% Gammaproteobacteria (**Figures 6B, C**). Three OTUs, Sutterellaceae, Enterobacteriaceae, and *Escherichia*, accounted for 98% of reads in 4 of 5 VANC-treated mice (**Figure 6D**). VANC-treated MN mice also had an average of 0.02% prevalence of Bacteroidetes and 0.64% Firmicutes compared to 12% and 78.07% in untreated MN mice. Collectively, these data indicate that COL treatment was successful in depleting Gammaproteobacteria and Betaproteobacteria, and VANC treatment led to an overwhelming expansion of the Gammaproteobacteria.

To determine if the antibiotic-mediated change in cecal microbiota composition modulated the systemic inflammatory response, we challenged MN mice intradermally with LPS as described previously. COL-treated MN mice had similar levels of inflammatory cytokines compared to control MN mice (**Figure 6E**). However, VANC-treated MN mice, which showed dramatic expansion of the Gammaproteobacteria, had significantly increased LPS-induced expression of *Il1b*, *Il6*, *Cxcl1*, and *Il17a* compared to either control or COL-treated MN mice.

We also evaluated how the oral antibiotics impacted bacterial translocation. Consistent with our previous finding that most culturable bacteria translocated to the liver and spleen were gram-positive species, VANC-treated mice had significantly reduced levels of culturable bacteria in the spleen and liver compared to COL-treated MN mice (**Figure 6F**). Conversely, translocation of bacteria to liver and spleen in COL-treated mice was not different from controls. Lastly, we examined weight change in mice over the two-week course of antibiotics to see how altered intestinal microbiota composition impacted body weight. While VANC-treated MN mice, which had expansion of the gut Proteobacteria, exhibited similar weight loss to control (untreated) MN mice, COL-treated mice, which had depletion of the Gammaproteobacteria, were able to maintain their body weight on the nutrient deficient diet compared to the control and VANC-treated MN mice (**Figure 6G**). Collectively, the data suggest that the antibiotic-mediated modulation of inflammation and weight loss was independent of a change in translocation of bacteria.

## Discussion

The pathogenesis of acute malnutrition and the mechanism(s) that drive a child’s increased risk of infection and death remain obscure (2). In this study, we used an established mouse model of acute weanling malnutrition that mimics childhood MAM to investigate the interplay between undernutrition, gut microbiota, intestinal barrier function, and systemic inflammation. We found that MAM leads to chronic inflammation in the absence of infection and an exaggerated inflammatory response to bacterial products. This occurred in the setting of increased translocation of bacteria and bacterial LPS from the gut. Strikingly, intestinal inflammation and barrier dysfunction was prominent in the large intestine but not in the small intestine (ileum). An increased proportion of Gammaproteobacteria in the cecum of MN mice, which was associated with increased total fecal LPS bioactivity, appeared to drive the inflammatory phenotype. Administration of non-absorbable antibiotics that reduced (colistin) or expanded (vancomycin) intestinal Gammaproteobacteria modulated the systemic inflammatory response and weight loss in malnourished mice.

The hyper-inflammatory phenotype we observed in malnourished mice recapitulates our findings and those of others in children with MAM (25, 36). It contrasts with findings in children with SAM, who typically display decreased circulating levels of inflammatory cytokines at baseline and a reduced inflammatory response to infection (3, 7). Thus, there appears to be a spectrum of inflammatory responsiveness that is inversely proportional to the level of undernutrition. These results suggest that in moderate malnutrition, immune cells are primed to have greater production of cytokines. A previous study of malnourished mice observed that interepithelial lymphocytes could be primed by a consortium of intestinal pathobionts to secrete inflammatory cytokines after stimulation (16), supporting the notion that immune cells may be primed for a more vigorous response by changes in the intestinal microbiota. This chronic immune stimulation may contribute to poorer outcomes from infection due to reduced disease (tissue) tolerance (37, 38) and exacerbated wasting due to increased energy cost (22).

The underlying mechanism driving the apparent immune priming is uncertain, but is likely due to chronic, low level exposure to bacterial LPS or other bacterial products. Children with severe acute malnutrition have elevated circulating LPS and evidence of endotoxin tolerance (39), but they also have more severe disruption of intestinal architecture that is not common to MAM (24). Another study found that moderately underweight infants (weight-for-age Z score <-2, >-3) had elevated EndoCAb IgG, a marker of increased exposure to circulating endotoxin, that correlated with increased intestinal permeability (40). In contrast to the induction of endotoxin tolerance following exposure to high levels of LPS, exposure of cells to low levels of LPS can induce endotoxin priming such that the inflammatory response to a second exposure is exaggerated (41). The exaggerated inflammatory response to *in vivo* LPS challenge and the *ex vivo* response of peritoneal cells to LPS and LTA challenge are consistent with endotoxin priming (41), and possibly cross-priming from other bacterial products (42). Furthermore, chronic exposure to low level of LPS my lead to epigenetic changes that modify (in this case exaggerate) the leukocyte response to an inflammatory stimulus (43). This “innate immune memory” may be a protective adaptation to reduce risk of impending infectious challenges, but could put the host at risk of inflammation-mediated tissue damage and increase severity of bacterial sepsis.

Understanding the site of intestinal barrier dysfunction in acute malnutrition is critically important. We first examined the histological structure of the ileum, cecum and colon. We found no significant differences in the villous height, inflammation, or other histologic features in the ileum. However, the large intestine (colon and cecum) was significantly shortened (an indicator of inflammation) had significantly reduced crypt depths, increased number of cells lining the crypts, increased inflammatory infiltrate in the lamina propria, and increased MPO expression. In a slightly different model of acute malnutrition (low-protein, low-fat diet) (28) ileal villi were blunted in MN males but not MN females. Oral gavage of mice with low and medium molecular weight fluorescent dextran did not show increased paracellular translocation in MN mice at a time when the dextran would have likely transited to the ileum. However, the report of reduced intestinal mobility in MN mice (28) suggests these data need to be interpreted cautiously. On the other hand, intracolonic delivery of low molecular weight fluorescent dextran revealed increased paracellular translocation in the colon in MN mice. These findings were confirmed by *ex vivo* studies of intestinal epithelium in Ussing diffusion chambers that showed dramatically increased paracellular permeability of the colon and cecum but not the ileum. Collectively, these data suggest impaired intestinal barrier function of the cecum and colon, which is contrary to the prevailing thought that barrier dysfunction in the small intestine is of primary importance in malnutrition. The previous focus on the small intestine has understandably been driven by the primary importance of the small intestine in nutrient absorption. Its plausibility is further supported by clinical and epidemiological overlap between chronic malnutrition (stunting) and environmental enteric dysfunction (EED), and clinical investigation of children with SAM and EED that show serum markers of intestinal barrier dysfunction and significant structural alteration in biopsies of the small intestine obtained through upper endoscopy. We are not aware of any evaluation of the colon in children with EED/stunting or acute malnutrition. Studies in mouse models of undernutrition (protein deficiency) have also focused on the small intestine where a range of severity in histopathological changes, increased intestinal permeability, and altered expression of tight junction proteins and claudins have been shown to be dependent on the level of protein restriction (17). A single study reported increased permeability in duodenum and proximal colon (but not distal colon) in MN male but not female mice (28). None of these studies investigated the impact of undernutrition on the large intestine.

The implication that the large intestine as the likely source of LPS translocation in our study is consistent with the known distribution of commensal bacteria in the gut. While the contents of the ileum contain 10^7^-10^8^ culturable bacteria per mL (44), and translocation can occur without overt histopathological changes, the number of bacteria in the colon is 5-6 orders of magnitude greater. Furthermore, it is estimated that >99% of bacterial LPS in the gut is found in the colonic lumen (45). Our finding of increased paracellular translocation of small molecules is likely an indicator of inflammation and impaired barrier function, but a paracellular route of LPS translocation seems unlikely since luminal LPS often exists as high molecular weight aggregates or is incorporated into mixed micelles, both of which would be too large to cross the epithelium through paracellular spaces. Our finding of increased peripheral blood LPS relative to portal blood LPS in malnourished mice suggests that translocation of LPS across the colonic epithelium occurs through a mechanism different from that in the normal host, or that there is impaired removal and detoxification of the LPS (whether it came from portal or peripheral blood) as it passes through the mesenteric lymph nodes or hepatic sinusoids (46). In our previous study, the skin-draining LN of malnourished mice exhibited reduced numbers of phagocytic cells, diminished barrier function, and increased dissemination of an intracellular pathogen (47). The barrier capacity of mesenteric LNs and defense against translocation of bacteria and bacterial LPS may be similarly compromised. The liver is also critical for removal of bacteria and LPS from circulation and failure of this firewall can result in increased systemic inflammation (48, 49). Undernourished neonatal mice develop liver injury, autophagy, oxidative stress, inflammation, and altered bile acid metabolism that undoubtedly affect immunological function in this organ (50). Protein-deprived adult mice exhibited reduced number and proliferation of Kupffer cells (51). Thus, reduced barrier function in the MLN and liver would amplify the effect of increased LPS translocation and consequent inflammation in MN mice. Further investigation into the mechanism(s) of malnutrition-related chronic endotoxemia is needed to understand the pathophysiology that underpins this disorder.

Gut microbiota play a critical role in modulating intestinal integrity and immune function. Studies of dysbiosis in malnourished children differ depending on the geographic location, age group, local diet, and other factors. However, loss of microbial diversity and altered microbiota composition (decreases in Bacteroidetes, and increases in Proteobacteria) are common themes (12, 14, 52, 53). Altered microbiota composition and beta diversity were recapitulated in our model. Loss of bacteria in the Bacteroidetes phylum, such as those in genus *Bacteroides*, indicate a loss of commensals that contribute to enterocyte health through fermentation of soluble fiber and production of short chain fatty acids (SCFA) and Vitamin K. Expansion of Firmicutes genera such as *Enterococcus* and Proteobacteria genera such the *Escherichia*/*Shigella* group (as seen in this study) is also commonly observed in malnutrition (12) and has been linked to development of intestinal and systemic inflammation (21). The pathophysiological role of altered microbiota and increased circulating LPS is strikingly reminiscent of what is seen in obesity, at the opposite pole of the malnutrition spectrum (20, 54). Proteobacterial blooms also occur concurrently with the development of colitis in a variety of mouse models and are over-represented in the intestinal microbiota of humans with metabolic disorder and inflammatory bowel disease (53). Furthermore, LPS from Proteobacteria and Bacteroidales have different effects on TLR4 signaling (29). LPS from Bacteroidales species comprises the majority of fecal LPS in a healthy person and dampens TLR4 signaling by competing with highly bioactive LPS from the Proteobacteria (29). Indeed, we found that MN mice had a more bioactive fecal LPS mass than control mice. Furthermore, the decrease in *E. coli* and rise in *Bacteroides* in colistin-treated mice was accompanied by decreased systemic inflammatory response and reduced weight loss. Conversely, the dramatic expansion of Gammaproteobacteria, which bear highly-proinflammatory LPS, and loss of *Bacteroides*, with anti-inflammatory LPS (29), in VANC-treated mice was associated with increased energetically-costly LPS-induced inflammation. Since there was decreased translocation of live bacteria to the spleen and liver in VANC-treated mice (because VANC eliminated gram-positive aerobes, which comprised the majority of the translocated bacteria), translocated bacteria are not likely to be the primary cause of the systemic inflammation and immune priming.

Several mechanisms could contribute to the amelioration of weight loss in colistin-treated MN mice. Compared to untreated MN mice, colistin-treated MN mice had greater proportions of bacterial genera known to enhance nutrient harvesting (*Bacteroides*, *Parabacteroides*, *Butyvibrio*) (55) and reduced proportions of Gammaproteobacteria that have been associated with malnutrition (12) and inflammation (19). Additionally, colistin’s ability to bind and inactivate LPS (56) may directly reduce exposure to LPS in the intestine and its translocation into the circulation. Depletion of bacteria with highly bioactive LPS may also reduce immune activation (29) and the consequent energy consumption (57, 58). The reduced weight loss of colistin-treated mice occurred despite increased translocation to the spleen and liver, again suggesting that translocated gram-positive bacteria do not play a primary role in the inflammation and weight loss of malnutrition.

Several limitations of this work should be considered. First, although this model of protein-energy and micronutrient malnutrition has features that represent childhood MAM (32), it does not incorporate other environmental stressors that commonly contribute to MAM in children. In particular, exposure to enteric pathogens is likely to amplify intestinal barrier dysfunction and systemic inflammation. Second, our evaluation of bacterial burden in the MLN, spleen, and liver by culture of tissue homogenates is incomplete since non-culturable bacteria would have been missed (59). Third, we utilized a highly sensitive TLR4-dependent reporter bioassay for measurement of LPS concentration, but other bacterial products may also signal through TLR4 and could be detected.

In summary, our work describes a state of baseline systemic inflammation and heightened systemic inflammatory response in a model of MAM that occurs in the context of intestinal dysbiosis, colonic inflammation and barrier dysfunction, and increased translocation of bacteria and bacterial LPS. Modulation of the intestinal Gammaproteobacteria community in the intestinal microbiota with antibiotics had striking effects – an increase exacerbated inflammation and a reduction ameliorated weight loss. Therefore, targeting selected intestinal bacterial communities may benefit children with malnutrition by interrupting the pathophysiological cascade of intestinal dysbiosis, impaired intestinal barrier, and systemic inflammation that lead to growth faltering. Furthermore, shifting the focus of monitoring and treatment programs away from simple growth metrics to the identification and targeting of early pathophysiologic markers will promote more rational therapies and improve long term recovery from childhood MAM. Further exploration into these mechanisms and potential therapies should be pursued as we work towards better treatment of childhood malnutrition.

##  Data Availability Statement

The original metagenomic contributions presented in the study are publicly available. These data can be found here: https://www.ncbi.nlm.nih.gov/sra/PRJNA812855. All other original contributions presented in the study are included in the article/Supplementary Material. Further inquiries can be directed to the corresponding author.

## Ethics Statement

The animals used in this study were handled in strict accordance with the recommendations in the Guide for the Care and Use of Laboratory Animals of the National Institutes of Health. The protocol was approved by the Institutional Animal Care and Use Committee of the University of Texas Medical Branch, Galveston, Texas (protocol number 1306027).

## Author Contributions

GTP, PM, SD, EO, and GAP conceived the project and designed the experiments. GTP, EO, AP, EC, ML, NA, II, and JS performed the experiments. GTP, EYO, EC, AP, NJA, II, GAP, JS, and PM analyzed the data. GTP wrote the initial manuscript with all authors providing critical feedback. BT, OS, SD, and GAP contributed fruitful discussions and helpful ideas. GTP, EO, PM, GAP, and BT made critical revision of the manuscript. All authors contributed to the article and approved the submitted version.

## Funding

This work was supported by the U.S. National Institutes ofHealth (NIH/NIAID) grant number AI130126 to PM. GTP was supported by NIH/NIAID training grant number 2T32AI007526. GAP was supported by NIH/NIDDK grant number K08DK113114 and P30DK56338 which funds the Texas Medical Center Digestive Diseases Center.

## Conflict of Interest

The authors declare that the research was conducted in the absence of any commercial or financial relationships that could be construed as a potential conflict of interest.

## Publisher’s Note

All claims expressed in this article are solely those of the authors and do not necessarily represent those of their affiliated organizations, or those of the publisher, the editors and the reviewers. Any product that may be evaluated in this article, or claim that may be made by its manufacturer, is not guaranteed or endorsed by the publisher.

## Supplementary Material

Supplementary Table 1 qRT-PCR primers used in this study.

Supplementary Figure 1 Representative growth curves of control (Con) and malnourished (MN) mice. Groups of 5 mice were weighed and randomly assigned to the nutrient-deficient or control diet after weaning (Day 0). Weights were determined weekly and shown is the mean and standard deviation (left panel) and the mean and standard deviation of the percent change from the starting weight (right panel).

Supplementary Figure 2 Cytokine mRNA expression in skin of control (Con; n=5) and MN mice (n=5) 24 hrs after intradermal delivery of **(A)** sterile PBS (20 µL) or **(B)** Lipotechoic Acid (LTA; 50 µg in 20 µL). Determined by qRT-PCR. Mean expression in skin of control mice following PBS delivery was used as baseline to determine fold change. (*p<0.05; ** p<0.01; *** p<0.001).

Supplementary Figure 3 **(A)** Numbers of culturable anaerobic bacteria (Colony Forming Units; CFU) in the MLN, spleens, and livers of control (Con) and malnourished (MN) mice (n=5 mice per group). Data are presented as the median with interquartile range. **(B)** Percentage of translocated culturable aerobic and anaerobic bacteria by Genus that were isolated from liver and spleen of MN mice. A total of 67 colonies were randomly selected for identification by MALDI-TOF mass spectrometry.
