# Supplementary material for: Pathologic Inflammation in Malnutrition Is Driven by Proinflammatory Intestinal Microbiota, Large Intestine Barrier Dysfunction, and Translocation of Bacterial Lipopolysaccharide
Source: Front Immunol. 2022 May 26;13:846155. doi: 10.3389/fimmu.2022.846155 (PMC9204284; doi:10.3389/fimmu.2022.846155)
Supplement: Supplementary Table 1 — qRT-PCR primers used in this study. [file Table_1.pdf]

Supplementary Table 1 qRT-PCR primers used in this study

Target	Primer	Sequence (5’-3’)
18S	18S For	TTAGAGTGTTCAAAGCAGGCCCGA
	18S Rev	TCTTGGCAAATGCTTTCGCTCTGG
Ccl2	CCL2 For	GCTCAGCCAGATGCAGTTAACGC
	CCL2 Rev	TGGGGTCAGCACAGACCTCTCT
Cxcl1	CXCL1 For	ATCCAGAGCTTGAAGGTGTTG
	CXCL1 Rev	GTCTGTCTTCTTTCTCCGTTACTT
Cxcl2	CXCL2 For	CCAGACAGAAGTCATAGCCACT
	CXCL2 Rev	GGTTCTTCCGTTGAGGGACA
Il1b	IL1B For	TTGACGGACCCCAAAAGATG
	IL1B Rev	AGAAGGTGCTCATGTCCTCAT
Il17a	IL17A For	ACCAGCTGATCAGGACGCGC
	IL17A Rev	CCAGGCTCAGCAGCAGCAACA
Il6	IL-6 For	AGACAAAGCCAGAGTCCTTCAGAGA
	IL-6 Rev	GCCACTCCTTCTGTGACTCCAGC
Tnf	TNF For	AGCCGATGGGTTGTACCTTGTCTA
	TNF Rev	TGAGATAGCAAATCGGCTGACGGT
Nos2	NOS2 For	TGGCTCGCTTTGCCACGGAC
	NOS2 Rev	GCTGCGACAGCAGGAAGCCA
Mpo	MPO For	CAGCGAGGACCCCCTAGCCA
	MPO Rev	GGCATCTCGCTGGAGCGCAT
Ifng	IFNg For	GCAACAGCAAGGCGAAAAAGGA
	IFNg Rev	TGAATGCTTGGCGCTGGACCTG
