# Supplementary figures and images for: Pathologic Inflammation in Malnutrition Is Driven by Proinflammatory Intestinal Microbiota, Large Intestine Barrier Dysfunction, and Translocation of Bacterial Lipopolysaccharide
Source: Front Immunol. 2022 May 26;13:846155. doi: 10.3389/fimmu.2022.846155 (PMC9204284; doi:10.3389/fimmu.2022.846155)

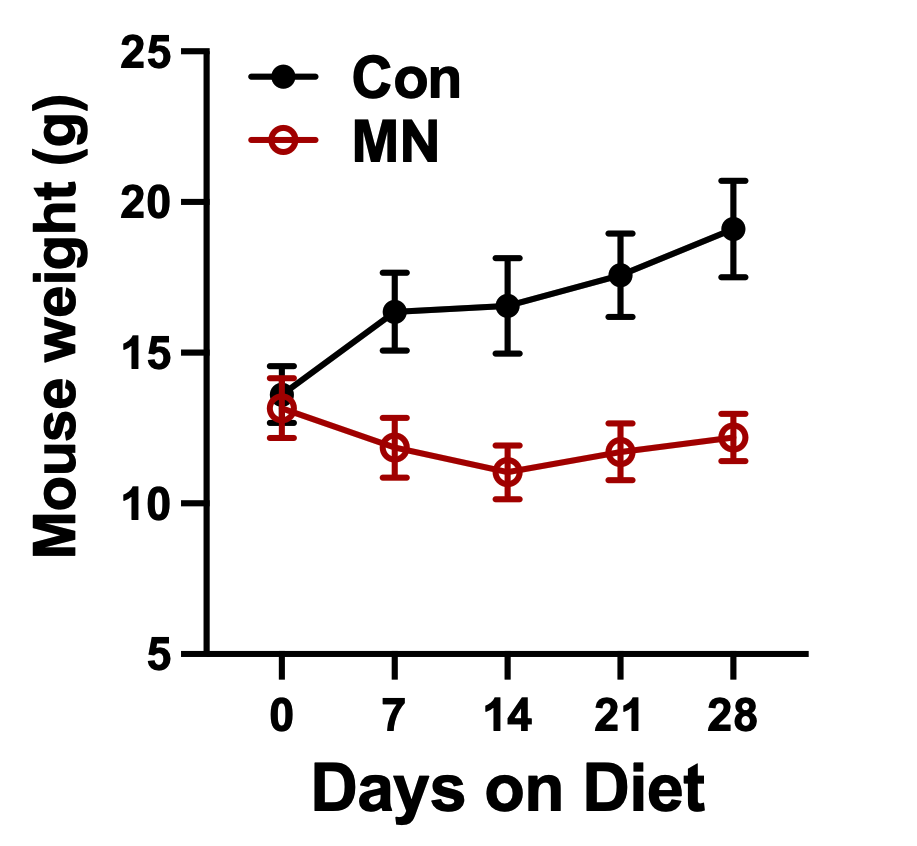

Supplement: Supplementary Figure 1 — Representative growth curves of control (Con) and malnourished (MN) mice. Groups of 5 mice were weighed and randomly assigned to the nutrient-deficient or control diet after weaning (Day 0). Weights were determined weekly and shown is the mean and standard deviation (left panel) and the mean and standard deviation of the percent change from the starting weight (right panel). [file Image_1.tiff]

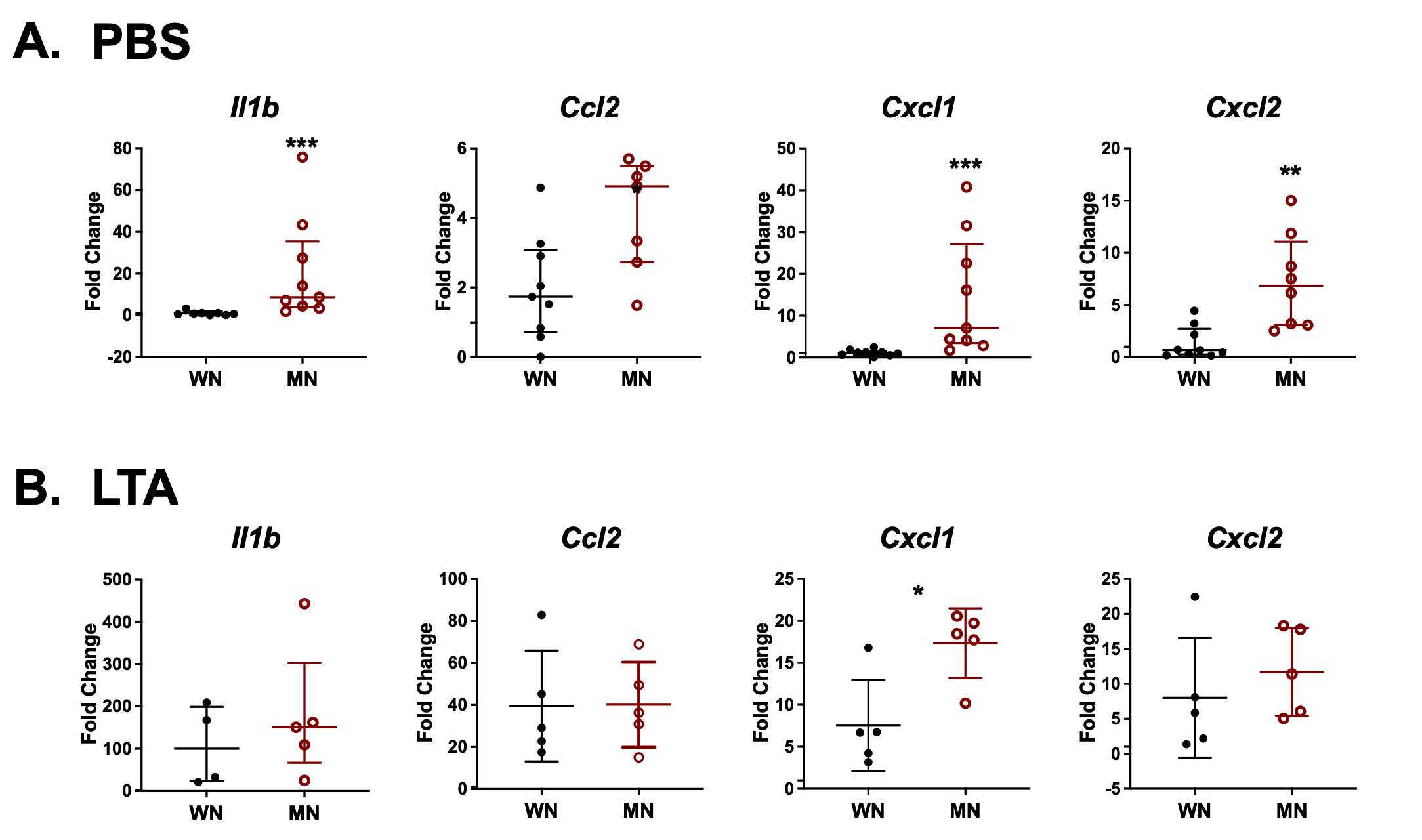

Supplement: Supplementary Figure 2 — Cytokine mRNA expression in skin of control (Con; n=5) and MN mice (n=5) 24 hrs after intradermal delivery of (A) sterile PBS (20 µL) or (B) Lipotechoic Acid (LTA; 50 µg in 20 µL). Determined by qRT-PCR. Mean expression in skin of control mice following PBS delivery was used as baseline to determine fold change. (*p<0.05; ** p<0.01; *** p<0.001). [file Image_2.tiff]

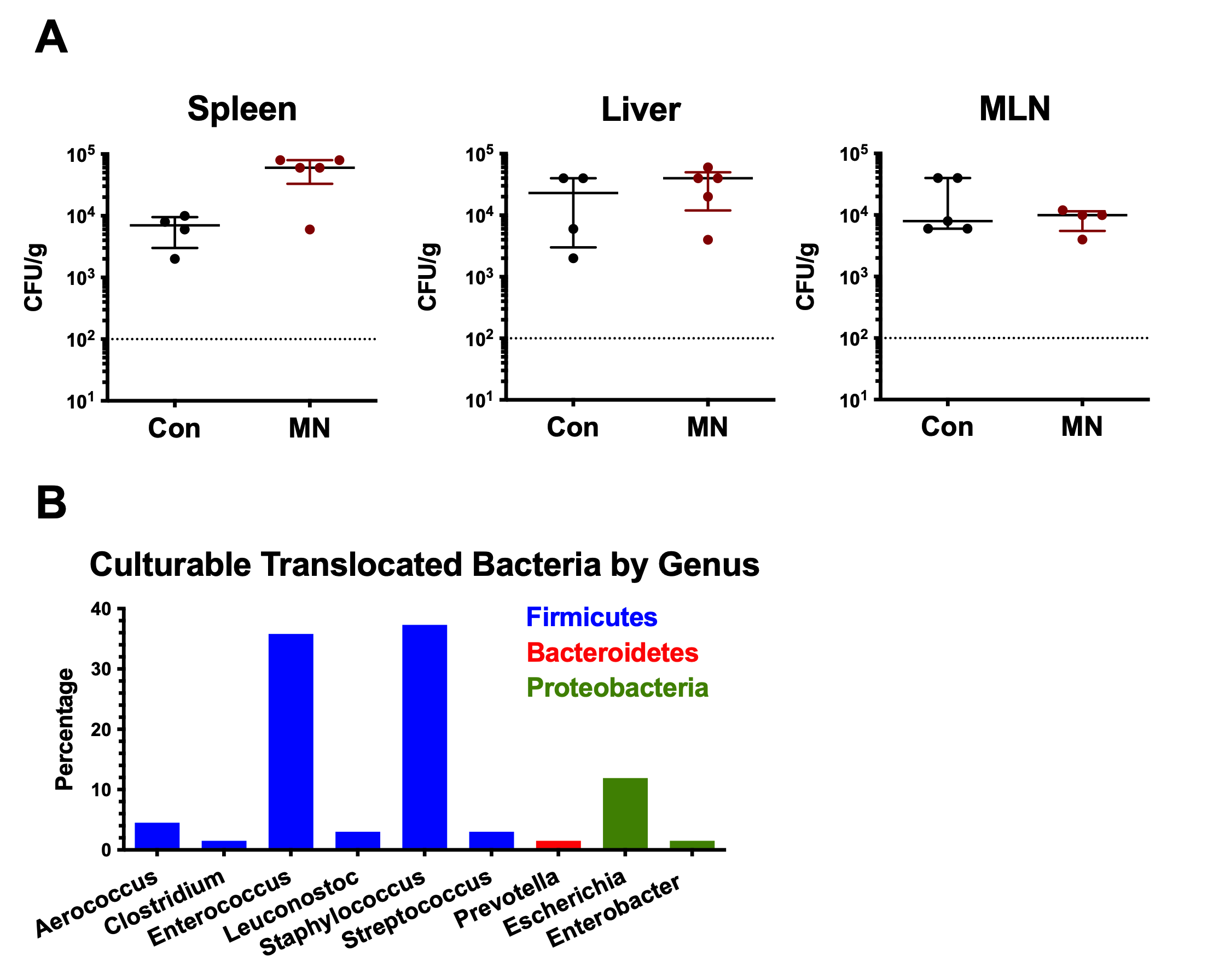

Supplement: Supplementary Figure 3 — (A) Numbers of culturable anaerobic bacteria (Colony Forming Units; CFU) in the MLN, spleens, and livers of control (Con) and malnourished (MN) mice (n=5 mice per group). Data are presented as the median with interquartile range. (B) Percentage of translocated culturable aerobic and anaerobic bacteria by Genus that were isolated from liver and spleen of MN mice. A total of 67 colonies were randomly selected for identification by MALDI-TOF mass spectrometry. [file Image_3.tiff]
